# Augmented Reality Ultrasound-Guided Vascular Access via Apple Vision Pro

**DOI:** 10.1016/j.jscai.2026.105452

**Published:** 2026-06-09

**Authors:** Jack Miller, Tobias Bexten, Stefan Wiebe, Matthew Read, Trevor Simard, Suraj Yalamuri

**Affiliations:** a Department of Cardiovascular Medicine, Mayo Clinic, Rochester, Minnesota; b Intensive Care Unit, Helios Dr Horst Schmidt Clinic Wiesbaden, Wiesbaden, Germany

## Introduction

Ultrasound (US)-guided vascular access affords several advantages over palpation-based techniques, including a decrease in first-attempt failure, mean time to success, and a reduction in hematoma complications.^1^ However, the location of the procedure site is often discrepant from that of the visual display, adversely impacting safety, efficacy, and ergonomic issues.^2^ Augmented reality (AR) head-mounted displays (HMDs) may alleviate this disconnect by unifying the US image within the same working field of view.^3^^,^^4^

Augmented reality for US guidance has been reported with a monocular HMD to achieve vascular access with excellent first-attempt success and no access-related complications.^3^ Optical see-through AR in phantoms improved the vascular access completion time for both experts and novices.^4^ Despite these promising results, both solutions can degrade the US image quality although inadequate resolution and real-world lighting conditions.^5^ The primary objective of this study was to explore an alternative “passthrough” AR technique, video see-through, in which the Apple Vision Pro (AVP) (Apple Inc) is used to display the ultrasound imaging within the same view as the procedure site to mitigate these issues. Initial feedback was gathered from 21 participants to better understand potential benefits and limitations of this approach.

## Methods

This study was deemed exempt by the Mayo Clinic Institutional Review Board, with the use of animals approved by the University of Otago’s Animal Ethics Committee. We evaluated 21 physicians performing a vascular access training exercise. Feedback was collected through an anonymous survey that included a single net promoter score (NPS) question along with additional open-ended questions and ideas for improvement. The NPS question is a 10-point Likert scale questionnaire that asks the user how likely they are to recommend a product or service to a friend or colleague. Scores of 1 to 6 indicate detractors, 7 to 8 indicate passives, and 9 to 10 indicate promotors. The responses from the open-ended feedback were grouped into themes, and the number of comments was noted.

**Figure 1:**
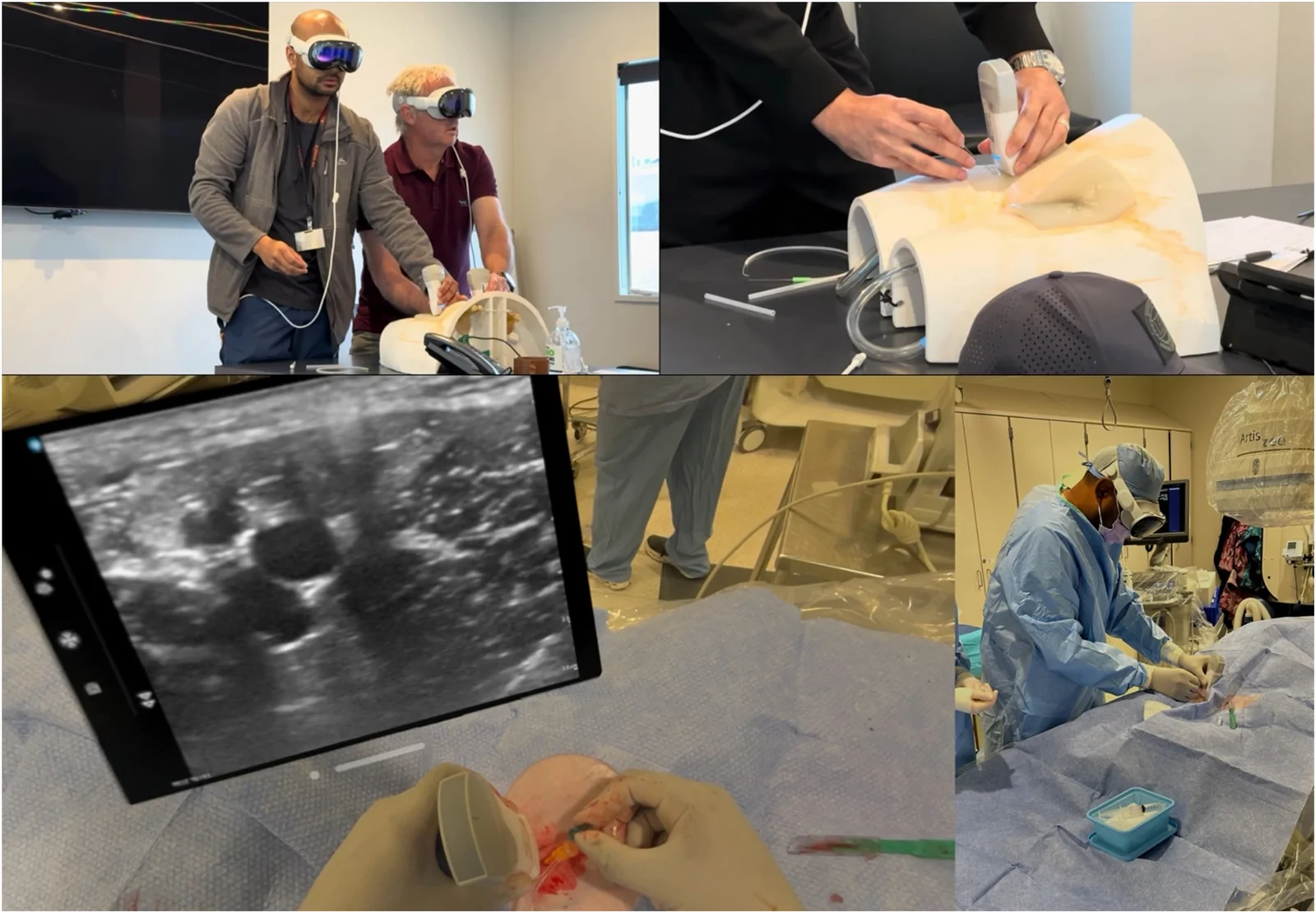
**Participants using the Apple Vision Pro with aVscan(GE HealthCare)on a phantom (top) and animal (bottom).**

The AVP was used for the display device. A Vscan (GE HealthCare) was used as the US probe, with the iOS Vscan Air application being compatible with the AVP, facilitating a direct wireless connection between devices. The study was performed at an extracorporeal membrane oxygenation medical symposium at the University of Otago in New Zealand with physicians (anesthesiologists, intensivists, and cardiac surgeons) from around the world. The primary task of this study was to use the AVP and Vscan to gain vascular access in both a live animal setting and a phantom setting (Figure 1).

## Results

Feedback was collected from a total of 21 participants. Through the NPS survey, it was found that there were 10 (48%) detractors, 6 (29%) passives, and 5 (24%) promoters, resulting in a final NPS of −24 with an average rating of 6.24. The common themes regarding what participants liked included the quality of the US image (7 [33%]), the ability to move and scale the user interface (5 [24%]), the lack of large physical equipment (5 [24%]), the general concept (4 [19%]), and the simplicity of use (4 [19%]).

The most common theme regarding what participants disliked was the setup and calibration of the system (8 [38%]). This was expected, as the AVP requires participants to complete the full calibration process. Other issues included interacting with the user interface (6 [29%]), the quality of the real-world image (4 [19%]), a lack of mirrored view (3 [14%]), simulator sickness (2 [10%]), the US feed occluding the real-world view (2 [10%]), battery concerns (2 [10%]), and cost (1 [5%]). Improvements suggested included overlaying the US image on the patient’s anatomy, improving real-world image quality, controlling the US probe itself, and improving needle visualization.

## Discussion and Conclusions

The AVP-guided US access was promising, with 11 (52%) participants reporting at least neutral or positive experiences. The primary noted benefits include quality, interactions, and the removal of bulky equipment such as US carts and monitors. Although negative factors were reported, those who rated the solution the lowest still noted very positive views of the concept and its potential should these technical factors be addressed.

The most common concern noted was the system setup. This may be mitigated in practice by each operator being assigned their own device. There is further opportunity to streamline the setup process to ensure seamless integration. The second item of concern was related to a real-world passthrough image. Specifically, although the desire for improved visual quality was noted, the primary issue was related to virtual content overlaying real-world objects of interest (ie, images obscuring the view of the needle at the insertion site). Strategies to improve this include automating the image to points of the visual field, reducing virtual content opacity, and/or augmenting the opacity of real-world objects. Participants suggested that including controls on the probe and spatially registering the US image relative to the probe could prove beneficial.

Implementation may present various barriers. The base cost of the AVP is $3499, which may not be feasible for departments with limited funding. Additionally, the AVP must be compliant with the information technology infrastructure. Lastly, sanitation standards would need to be considered as the AVP is not designed for sanitization. However, in most use cases, cleaning the device would likely be sufficient.

This study was subject to various limitations. There was no comparison group to analyze the results of the study against. Further, the sample size was relatively small with only 21 participants. Finally, no objective measures were captured and the study focused entirely on the subjective experience of the participants. However, the intent of the study was not to present generalizable results but to demonstrate a proof of concept and gather initial feedback.

Overall, US-guided vascular access utilizing a binocular HMD with AR video-see-through capability may offer a promising approach for consolidating visual inputs into an operator’s field of view. This approach may translate well for other US-guided procedures where it is beneficial to see the procedure site and the US image within the same view. Future work will explore the implementation of this technique into practice as well as randomized controlled studies to compare against existing techniques, the inclusion of additional imaging modalities, and the potential benefits for novices.

## CRediT authorship contribution statement

**Jack Miller:** Writing – review & editing, Writing – original draft. **Tobias Bexten:** Writing – review & editing, Writing – original draft. **Stefan Wiebe:** Writing – review & editing, Writing – original draft. **Matthew Read:** Writing – review & editing, Writing – original draft. **Trevor Simard:** Writing – review & editing, Writing – original draft. **Suraj Yalamuri:** Writing – review & editing, Writing – original draft.

## Declaration of competing interest

The authors declared no potential conflicts of interest with respect to the research, authorship, and/or publication of this article.
